# Mushroom Lectins: Specificity, Structure and Bioactivity Relevant to Human Disease

**DOI:** 10.3390/ijms16047802

**Published:** 2015-04-08

**Authors:** Mohamed Ali Abol Hassan, Razina Rouf, Evelin Tiralongo, Tom W. May, Joe Tiralongo

**Affiliations:** 1 Institute for Glycomics, Griffith University, Gold Coast Campus, Gold Coast, QLD 4222, Australia; E-Mails: ali.abolhassan@griffithuni.edu.au (M.A.A.H.); r.rouf@griffith.edu.au (R.R.); 2 School of Pharmacy and Griffith Health Institute, Griffith University, Gold Coast Campus, Gold Coast, QLD 4222, Australia; E-Mail: e.tiralongo@griffirh.edu.au; 3 Royal Botanic Gardens Melbourne, South Yarra, VIC 3141, Australia; E-Mail: Tom.May@rbg.vic.gov.au

**Keywords:** lectins, mushrooms, bioactivity, structure, antiproliferative activity, immunomodulatory activity, antiviral activity

## Abstract

Lectins are non-immunoglobulin proteins that bind diverse sugar structures with a high degree of selectivity. Lectins play crucial role in various biological processes such as cellular signaling, scavenging of glycoproteins from the circulatory system, cell–cell interactions in the immune system, differentiation and protein targeting to cellular compartments, as well as in host defence mechanisms, inflammation, and cancer. Among all the sources of lectins, plants have been most extensively studied. However, more recently fungal lectins have attracted considerable attention due to their antitumor, antiproliferative and immunomodulatory activities. Given that only 10% of mushroom species are known and have been taxonomically classified, mushrooms represent an enormous unexplored source of potentially useful and novel lectins. In this review we provide an up-to-date summary on the biochemical, molecular and structural properties of mushroom lectins, as well as their versatile applications specifically focusing on mushroom lectin bioactivity.

## 1. Introduction

Lectins are proteins, non-immunglobulinn nature that bind diverse sugar structures with a high degree of selectivity and stereospecificity without altering the covalent structure of any recognized glycosyl ligands [1,2]. Lectins play crucial roles in various biological processes such as cellular signaling, malignancy, scavenging of glycoproteins from the circulatory system, cell–cell interactions in the immune system, differentiation and protein targeting to cellular compartments [1,3,4] and, also, in host defence mechanisms, inflammation, and metastasis [5,6]. Generally lectins are able to agglutinate erythrocytes and are often referred to as hemagglutinins. However, this is an over simplification, since not all hemagglutinins are lectins. That is, not all hemagglutinins agglutinate erythrocytes through reversible binding of sugars found on the cell surface; only lectins possess this activity [7]. Lectins are widespread in distribution and have been isolated from bacteria, insects, plants seeds and roots, algae, body fluid of vertebrates, lower vertebrates, mammalian cell membranes and fungi [8]. Some viruses, including influenza, reo virus, and picorna virus, primarily use lectins to attach to host cells.

Among all the sources of lectins, plants have been most extensively studied, with notable examples including legume lectins, type 2 ribosome inactivating proteins, chitin-binding lectins, and monocot mannose-binding lectins [9,10,11,12,13]. These plant lectins function as the defensive system against phytopathogenic fungi and predatory animals [14,15], and they also play a role in the symbiotic relationship of plants with nitrogen fixing bacteria [16]. In animals, lectins perform a variety of biological functions, from cell adhesion to glycoprotein synthesis, as well as controlling the protein level in the blood. Some mammalian liver cell lectin receptors are believed to be responsible for the removal of certain glycoproteins from the circulatory system [17]. Animal lectins also regulate differentiation and organ formation [18], play a vital role in the migration of lymphocytes from the bloodstream into the lymphoid organs, as well as in metastasis of cancer cells [19] and drug targeting [8,20].

For thousands of years mushrooms have been recognized for their medicinal properties, nutritious value and importance in spiritual ceremonies [21,22]. There have been extensive clinical trials conducted in China and Japan in order to illustrate that a number of mushrooms have medicinal and therapeutic value for the treatment/prevention of cancer, viral diseases, hypercholesterolaemia, blood platelet aggregation, and hypertension [23]. Mushrooms are essentially macrofungi that can be seen with the naked eye in contrast to microfungi, and they exhibit a distinctive fruiting body that can be hypogeous or epigeous [24]. It is estimated that there are 140,000 mushroom species that belong to the phyla Ascomycota and Basidiomycota [25]. However, only 10% of these mushroom species are known and have been taxonomically classified, thus making them an enormous unexplored source of potentially useful substances, including lectins [26,27,28,29]. Among known mushrooms, there are around 2000 species that are edible and about 200 have traditionally been collected for food, medicine or other purposes [30].

In recent years mushroom lectins have attracted considerable attention due to their antitumor, antiproliferative and immunomodulatory activities [19,31,32,33,34,35,36,37,38]. In this review, we provide an update, which builds on a series of reviews on mushroom lectins [19,36,38,39,40,41], that gives an up-to-date summary of the biochemical, molecular and structural properties of mushroom lectins, as well as their versatile applications specifically focusing on mushroom lectin bioactivity.

## 2. Mushroom Lectins

Mushrooms express high levels of lectins as storage proteins, that are thought to have a potential role in defence, similar to plant lectins [36,42]. In addition, a significant role for lectins is emerging in relation to symbiotic associations between fungi and other organisms, such as in mycorrhizas and lichens, and in cell interactions with respect to flocculation, mycelial aggregation and mating [43,44]. Mushroom lectins isolated from different species vary in molecular masses, subunit number and carbohydrate specificity (Table 1) [36], but lectins with very different biochemical properties have also been isolated from a single species [45,46]. Moreover, lectins have been purified from different parts of the mushroom, including caps, stalks, and mycelia, and the expression levels may vary depending on fruit-body age [47] and season [40]. For example, the quantity of *Laccaria laccata* lectin is higher in adult mushrooms whereas the expression of lectin from *Amanita muscaria*, *Tricholomopsis rutilans* and *Lactarius rufus* is higher in young mushrooms. Similarly, we have reported on mushrooms collected in Australia, in which the expression of lectin varies with respect to environmental influences, such as season, location, and year, as well as depending on macroscopic properties, such as age and mycelia growth [7].

**Table 1:** Mushroom lectins and their specificity.

Source of Lectin (Current Species Names Given in Parentheses)	Specificity of Sugars/Glycoproteins *	Ref.
*Agaricus arvensis*	Inulin	[48]
*Agaricus bisporus*	GalNAc, Galβ1,3GalNAc (T antigen), sialyl-Galβ (ABL) ^‡^	[49,50,51,52]
*Agaricus bitorquis*	Lac	[7]
*Agaricus blazei* (*Agaricus subrufescens*)	Methyl *N*-acetyl-α-*o*-galactosaminide, GalNAc, BSM, asialo-BSM, fetuin, asialofetuin	[53]
*Agaricus campestris*	GalNAc, Gal, Suc	[54,55]
*Agaricus pilatianus*	Lac, GlcNAc, Glc, Rham	[47]
*Agrocybe aegerita* (*Cyclocybe aegerita*)	Lac, BSM, Glycophorin A, k-Casein, β-galactosides, Gal (AAL galectin), terminal non-reducing GlcNAc (AAL2)	[56,57,58,59]
*Agrocybe cylindracea* (*Cyclocybe cylindracea*)	Trisaccharides containing Neu5Acα2,3Gal, Lac, sialic acid, inulin (ACG)	[60,61,62,63]
*Aleuria aurantia*	l-Fuc, fucosyl oligosaccharides (AAL)	[64,65]
*Amanita muscaria*	*O*-type glycans	[66]
*Amanita ovoidea*	Gal, GalNAc, Rham	[47]
*Amantia pantherina*	GIcNAcβ1, 4ManβpNP, Galβ1,4GlcNAcβ1,4GIcNAc, Galβ1,4GIcNAcβ1,4GlcNAc, BSM, asialo-BSM	[67]
*Amanita phalloides*	Ovomucin, human glycophorin A (A. *phalloides* lectin)	[66]
*Amanita virosa*	Blood group specific substance B, A and H, bovine thyreoglobulin, ovomucoid, asialo-ovomucoid transferrin, Ovine submaxillary mucin, 4-nitrophenyl-α-d-mannopyranoside, 4-nitrophenyl-β-d-glucopyranoside, 4-nitrophenyl-β-d-galactopyranoside	[68]
*Amanita muscaria*	*O*-type glycans	[66]
*Armillaria luteovirens*	Inulin (ALL)	[69]
*Auricularia polytricha* (*Auricularia cornea*)	Raf, Gal, ovomucoid and β-anomers of galactoside (Lac, *p*-nitrophenyl β-d-galactoside)	[70]
*Boletopsis leucomelaena* [*as* “*leucomelas*”] ^#^	GlcNAcβ1,2Manα1,3(GlcNAcβ1,2Manα1,6)Manβ1,4GlcNAcβ1, 4GlcNAc, GlcNAc (BLL)	[71]
*Boletus edulis*	d(+)-Mel, d-Xyl (BEL)	[72]
*Boletus satanas*	d-Gal	[73]
*Boletus subtomentosus* (*Xerocomus subtomentosus*)	d-Lac	[74]
*Boletus venenatus*	Asialofetuin, Galβ1,4GlcNAcβ1,4Manβ1,4GlcNAcβ1,4GlcNAc residues in *N*-linked sugar chains	[75]
*Chlorophyllum brunneum*	Neu5Ac	[7]
*Chlorophyllum molybdites*	Neu5Gc, GalNAc, asialo-BSM, PSM	[76]
*Ciborinia camelliae*	GalNAc	[77]
*Clavaria purpurea* (*Alloclavaria purpurea*)	Asialo-BSM, α-Gal, Galα1,3Gal, Raf	[78]
*Clitocybe geophyla* ^	GalNAc, Lac, Glc	[47]
*Clitocybe nebularis*	Asialo-fetuin, Lac, GalNAc, Gal, *N*,*N*-diacetyllactosediamine (GalNAcβ1,4GlcNAc, LacdiNAc) (CNL)	[79,80,81]
*Coprinus atramentarius* (*Coprinopsis atramentaria*)	d-Lac	[74]
*Coprinus comatus*	GlcNAc, Lac, Gal, Ara, Rib, Xyl	[7,47]
*Coprinus cinereus* (*Coprinopsis cinerea*)	β-Gal (CCL2), GlcNAcβ1,4(Fucα1,3)GlcNAc (CGL2), GalNAcβ1,4GlcNAc (CGL3)	[82,83,84]
*Coprinus micaceus* (*Coprinellus micaceus*)	Lac, Gal, GalNAc	[47]
*Cordyceps militaris*	Sialoglycoprotein, Neu5Ac (CML)	[85]
*Cortinarius* sp. *TWM 1710*	Gal	[7]
*Flammulina velutipes*	β-d-Gal, fetuin, human transferrin, human glycophorin, lactoferrin (F. *velutipes* lectin)	[86,87]
*Fomes fomentarius*	GalNAc, α-d-Gal, Raf	[80]
*Ganoderma capense*	d-Gal, d(+)-Galactosamine (*G. capense* lectin)	[88]
*Ganoderma lucidum*	Asialo-triantennary *N*-glycan, *N*-and *O*-linked glycans.	[89,90]
*Grifola frondosa*	Terminal GalNAc residues, porcine stomach mucin, linear d-Rham, PSM (GFL)	[76,91,92]
*Hericium erinaceus* [*as* “*erinaceum*”] ^#^	Neu5Gc, Neu5Ac, inulin (HEA)	[62,93,94]
*Hygrophorus hypothejus*	Lac, d-Gal, d-GalNAc, Galβ1,4GlcNAc, *o*-nitrophenylα-d-GalNAc, *p*-nitrophenyl-β-d-GalNAc, asialo-BSM	[95,96]
*Hygrophorus russula*	α1,6-mannobiose, Isomaltose (Glcα1,6Glc), isomaltotriose, isomaltotetraose, isomaltopentaose, isomaltohexaose, methyl α-mannoside, α1,3-mannobiose, methyl β-mannoside, α1,2-mannobiose, α1,4-mannobiose, methyl α-glucoside, Man, lacturose (HRL)	[97]
*Inocybe fastigiata* (*Inocybe rimosa*)	GalNAc	[98]
*Inocybe umbrinella*	Raf, d-Mel, α-Lac, d-Gal (*I. umbrinella* lectin)	[99]
*Ischnoderma resinosum*	Methyl-β-galactoside, Fuc, l-Ara	[53]
*Kuehneromyces mutabilis*	Asialo-PSM, asialofetuin, fetuin, α1-acid glycoprotein, Ovomucoid	[100]
*Laccaria amethystina*	l-Fuc (LAF), d-Lac and GalNAc, BSM, asialo-BSM,PSM, asialo-PSM, human glycophorin A (LAL)	[101,102]
*Laccaria laccata*	l-Fuc	[103]
*Lactarius deliciosus*	Galβ1,3GalNAc	[104]
*Lactarius deterrimus*	Galβ1,3GalNAc	[105]
*Lactarius flavidulus*	d-Mel, d-Fru, l(+)-Rham, Sor, d-Gal, d(+)-Man, Lac, d(+)-Xyl, l(+)-Ara, d-Glu, Raf, Inulin, *p*-nitrophenyl-α,d-glucopyranoside, *p*-nitrophenyl-β,d-glucopyranoside, inositol (LFL)	[106]
*Lactarius lignyotus*	Asialofetuin, asialo-PSM and other desialylated glycoproteins	[107]
*Lactarius pergamenus*	GalNAc, 4-nitrophenyl-β-d-galactopyranoside, α-phenyl *N*-acetyl-d-glucosaminopyranoside, Bovine thyroglobulin, human transferrin, Orosomucoid (α-glycoprotein), sheep submaxillary mucin, BSM, asialo-BSM, fetuin	[108]
*Lactarius rufus*	α-phenyl *N-*acetyl-d-glucosaminopyranoside, 4-nitrophenyl-β-d-glucosamine, asialo-BSM, human and bovine thyroglobulin, group specific substances from human erythrocytes	[109]
*Lactarius salmonicolor*	Galβ1,3GalNAc	[110]
*Laetiporus sulphureus* [*as* “*sulfureus*”] ^#^	LacNAc, l-Rham, salicine, asialo-BSM,BSM, asialofetuin, lacto-*N*-*neo*tetraose (Galβ1,4GlcNAcβ1,3Galβ1,4Glc) (LSL)	[111,112,113]
*Lentinus edodes* (*Lentinula edodes*)	GlcNAc, GalNAc, Man, d-Mel, Gal	[114,115,116,117]
*Lentinus squarrosulus*	Raf, d-Suc, Rib	[118]
*Lepiota leucothites* (*Leucoagaricus leucothites*)	Glc, GlcNAc, Man	[47]
*Lepiota rhacodes* (*Macrolepiota rachodes*)	Lac, Arab, MethGLc, GalNAc	[47]
*Lepista nuda*	Gal,Fuc, Suc, Arab	[47]
*Lyophyllum decastes*	Galabiose-Galα1,4Gal, non-reducing α-Gal	[119]
*Macrolepiota procera*	Terminal *N*-acetyl-lactosamine, β-galactosides (MPL)	[120]
*Marasmius oreades*	Galα1,3Galβ1,4GlcNAc, blood group Btrisaccharide (Galα1,3Gal2,1αFuc), Man, thyroglobulin, asialofetuin, complex type *N*-glycans (MOA)	[121,122,123,124,125]
*Melanoleuca brevipes*	Gal, Rham, Lac	[47]
*Melastiza chateri*	l-Fuc	[126]
*Mycoleptodonoides aitchisonii*	Asialo-BSM, BSM	[127]
*Omphalotus nidiformis*	Lac, Gal, Ara, Rib	[7]
*Oudemansiella platyphylla* (*Megacollybia platyphylla*)	β-GalNAc, terminal GlcNAc	[47,76,128]
*Panus conchatus*	d-Gal	[40,129]
*Paxillus involutus*	Asialo-PSM, asialofetuin, fetuin, α1-acid glycoprotein (*P. involutus* lectin)	[103]
*Paecilomyes japonica* ^	Sialic acid and sialoglycoprotein (PJA)	[130]
*Peziza silvestris* [as “*sylvestris*”] ^#^ (*Peziza arvernensis*)	l-Ara (*P. silvestris* lectin)	[131]
*Phaeolepiota aurea*	GalNAc (PAL1 and PAL2)	[132]
*Phallus impudicus*	Fetuin	[76]
*Phlebopus marginatus*	Lac, Gal	[7]
*Pholiota adiposa*	Inulin (PAL)	[133]
*Pholiota aurivella*	Asialofetuin	[134]
*Pholiota squarrosa*	α1,6-fucosylated *N*-glycans	[135]
*Pleurocybella porrigens*	GalNAc, asialo-BSM, *O*-linked glycans	[136]
*Pleurotus citrinopileatus*	Mal, *o-*nitrophenyl-β-d-galactopyranoside, *o*/*p*-nitrophenyl-β-d-glucuronide, inulin (*P. citrinopileatus* lectin)	[137]
*Pleurotus cornucopiae*	Asialo-mucin	[138]
*Pleurotus eous*	Methyl-α-d-galactoside, galactosamine, mannosamine, asialofetuin (PEL)	[139]
*Pleurotus ostreatus*	Me-α-GalNAc and 2'-fucosyllactose (Fucα1,2Galβ1,4Glc), d-Mel, d-Gal, Raf, NeuNAc, Inulin, Lac, Galactosyl and *N*-Acetyl galactosaminyl groups, BSM, asialo-BSM (POL)	[32,140,141,142]
*Pleurotus serotinus* (*Sarcomyxa serotina*)	GalNAc	[129]
*Pleurotus spodoleucus*	Lac	[140]
*Pleurotus tuber-regium*	GlcNAc	[143]
*Polyporus adustus* [*as* “*adusta*”] ^#^ (*Bjerkandera adusta*)	d-Mel, d-Fru, d-Ara, d-Glu, d-Raf, *p*-nitro-α-d-glucopyranoside (*P. adustus* lectin)	[144]
*Polyporus squamosus*	Neu5Acα2,6Galβ1,4Glc/GlcNAc (6'-sialylated type II chain) of *N*-glycans (PSL)	[145,146,147,148]
*Psathyrella asperospora*	GlcNAc (PAL)	[7]
*Psathyrella velutina*	GlcNAc, Neu5Acα2,3Galβ1,4GlcNAc, Heparin and Pectin (PVL)	[62,149,150,151,152]
*Psilocybe barrerae*	d-Gal, Glycophorin, BSM, asialo-BSM, human serum and milk transferrin	[153]
*Russula delica*	Inulin, *o*-nitrophenyl-β-d-galactopyranoside (*R. delica* lectin)	[154]
*Russula lepida*	Inulin, *o*-nitrophenyl-β-d-galactopyranoside	[155]
*Russula nigricans*	Asialofetuin, asialo-PSM, fetuin, ovomucoid, α1-acid glycoprotein	[103]
*Schizophyllum commune*	GalNAc (*S. commune* lectin, species from Thailand), Lac (*S. commune* lectin, species from China)	[156,157]
*Stereum hirsutum*	l-Xyl	[158]
*Trametes versicolor*	Gal	[47]
*Tricholoma fracticum* [*as* “*fractum*”] ^#^	Lac, Gal, GalNAc	[47]
*Tricholoma mongolicum* (*Leucocalocybe mongolica*)	Lac (TML1), GalNAc and Gal (TML2)	[45]
*Volvariella volvacea*	Thyroglobulin (VVL)	[31]
*Xerocomus chrysenteron*	Asialofetuin, asialo-PSM and other desialyzed glycoproteins Sychrova, GalNAc, Gal,TF antigen (XCL)	[107,159,160]
*Xerocomus spadiceus* (*Xerocomus ferrugineus*)	Inulin (*X. spadiceus* lectin)	[161]
*Xylaria hypoxylon*	Inulin, Xyl (*X. hypoxylon* lectin)	[162]

* Ara, arabinose; BSM, Bovine submaxillary mucin; Fru, fructose; Gal, galactose; Glu, glucose; GalNAc, *N*-acetyl-d-galactosamine; GlcNAc, *N*-acetyl-d-glucosamine; Lac, lactose; Man, mannose; Mel, Melibiose; Neu5Gc, *N*-glycolyl-neuraminic acid; Neu5Ac, *N*-acetyl-neuraminic acid; PSM, Procine submaxillary mucin Raf, raffinose; Rham, rhamnose; Rib, ribose; Sor, sorbitol, Suc, sucrose; Xyl, xylose; **^#^** The species name was incorrectly spelt in the original publication (in square brackets), the correctly spelt name has now been provided; **^** Names could not be matched to the global fungi name databases. “*Clitocybe geophyla*” as reported by Mikiashvili *et al.* (2006) [47] could be “*Clitocybe geotropa*” or “*Inocybe geophylla*”. “*Paecilomyces japonica*” as reported by Park *et al.* (2004) [127] could be *Isaria japonica* Yasuda, in which case the current name is *Isaria tenuipes* following Luangsa-ard *et al.* (2005) [163]; ^‡^ These abbreviated lectin names are provided for those mushroom species that are mentioned in Section 4 and Section 5 and also for mushroom species that have two or more lectins.

The first mushroom lectin “phallin” was described in *Amanita phalloides*, a hemolytic agent [164]. Approximately 105 lectins have been identified in diverse mushroom species. Table 1 gives a complete list of lectins, thus far identified in mushroom, and their carbohydrate and/or glycoprotein specificity. The species names given in Table 1 have been updated (in parentheses) where appropriate predominantly using *Species Fungorum* (http://www.speciesfungorum.org/), and the spelling has also been corrected for some species names. Up-to-date nomenclature, especially in terms of correct generic placement, allows comparisons with phylogenetically related taxa and also facilitates targeted collecting of closely related species for the identification and isolation of novel lectins. Even though we have updated and corrected species names in Table 1, to avoid confusion we have used the originally species names (as published) from which the lectin(s) were identified in this review.

The largest number of lectins has been identified from *Lactarius* followed by *Pleurotus*, *Agaricus*, *Amanita* and *Boletus.* Interestingly, there are a number of mushroom species from which more than one lectin has been isolated, for example *Coprinus cinereus* [82,83,84], *Agrocybe aegerita* [56,57,58,59], *Agrocybe cylindracea* [60,61], *Laccaria amethystina* [101] and *Schizophyllum commune* [156,157]. Mushrooms species where multiple lectins have been identified, and those that are further discussed and listed in Table 2, Table 3 and Table 4, the published lectin abbreviations have been given in Table 1. As illustrated in Table 1, mushroom lectins have been identified with varying sugar specificities, from lectins that only bind the polysaccharide inulin (e.g., lectins from *Agaricus arvensis* [48], *Pholiota adiposa* [133], *Xerocomus spadiceus* [161]) to lectins that bind lactose (*Agaricus bitorquis*, *Boletus subtomentosus*, *Coprinus atramentarius*, *Pleurotus spodoleucus* [7,74,140]), galactose (*Boletus sataaus* and *Panus conchatus* [40,73,129]), *N*-acetylgalactosamine (*Ciborinia camelliae*, *Inocybe fastigiata*, *Lactarius vellereus*, *Pleurotus serotinus* [77,98,129]), and sialic acid (*Hericium erinaceus*, *Polyporus squamosus*, *Psathyrella velutina*, *Paecilomyes japonica*, and *Agrocybe cylindracea* [62]).

**Table 2:** Common structural characteristics and strategies used to purify mushroom lectins for which crystal structure data exists.

Lectin	Purification Strategy	Description of Lectin/Lectin Complex and Their PDB ID *	Resolution (Å)	Type of Fold ^#^, PDB Structure and ID	Similarity to Other Structures and Their PDB ID	Ref.
**Unique lectin-fold**						
*Lyophyllum decastes* lectin (LDL)	Melibiose-sepharose	LDL (Ligand free)/4NDS	1.00	Two β-sheets linked by disulfide bridges: 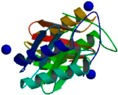 4NDS	Ginkbilobin-2/3A2E	[165]
		LDL globotriose complex/4NDV	1.00			
		LDL orthorhombic form/4NDT	1.30			
		LDL α-methylgalactoside complex/4NDU	1.03			
**β-propeller-fold**						
*Aleuria aurantia* lectin (AAL)	(NH_4_)_2_SO_4_ precipitation, fucose-starch AC ^	AAL (Ligand free)/1OFZ	1.50	Six-bladed β-propeller fold 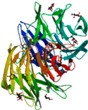 1OFZ	*A. fumigatus* Fuc-binding lectin (AFL)/4AGI	[76,166,167,168]
		AAL complexed with Fuc/1IUC	2.24			
		AAL (Hg-derivative from)/1IUB	2.31			
*Psathyrella velutina* lectin (PVL)	Chitin-sepharose AC, DEAE-cellulofine, CM-sepharose CL-6B	PVL (Ligand free)/2BWR	1.50	Integrin-like 7-blade β-propeller 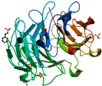 2BWR	*Phanerochaete chrysosporium* aldos-2-ulose dehydratase (AUDH)/4A7K	[76,149,150,152,169,170]
		PVL GlcNAc complex/2C4D	2.60			
		PVL Neu5Ac complex/2C25	1.80			
		PVL methyl 2-acetamido-1, 2-dideoxy-1-seleno-β-d-glucopyranoside complex/2BWM	1.80			
**Galectin-like fold**						
*Agrocybe aegerita* lectin/galectin (AAL-galectin)	(NH_4_)_2_SO_4_ precipitation, DEAE-sepharose FF, Sephacryl S-200 HR, GF-250 HPLC	AAL-galectin (Ligand free)/2ZGK	3.00	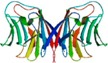 2ZGK	ACG complexed with blood type A antigen tetraose/3WG3	[171,172]
		Recombinant AAL-galectin (rAAL-galectin) (Ligand free)/2ZGL	1.90			
		rAAL-galectin Lac complex/2ZGM	1.90			
		rAAL-galectin Gal complex/2ZGN	2.50			
		AAL-galectin mutant H59Q Lac complex/2ZGO	2.00			
		AAL-galectin mutant I25G/2ZGP	2.70			
		AAL-galectin mutant L33A/2ZGQ	1.90			
		AAL-galectin mutant L33A/2ZGR	1.90			
		AAL-galectin mutant L47A/2ZGS	1.90			
		AAL-galectin mutant F93G/2ZGT	2.80			
		AAL-galectin mutant I144G/2ZGU	2.40			
		AAL-galectin TF antigen complex/3AFK	1.95			
		AAL-galectin *p*-nitrophenyl TF disaccharide complex/3M3C	2.00			
		AAL-galectin mutant E66A *p*-nitrophenyl TF disaccharide complex/3M3E	2.10			
		AAL-galectin mutant R85A *p*-nitrophenyl TF disaccharide complex/3M3O	2.10			
		AAL-galectin ganglosides complex GM1 pentasaccharide/3M3Q	2.10			
*Agrocybe cylindracea* galectin/lectin (ACG)	(NH_4_)_2_SO_4_ precipitation, DEAE-cellulofine A-200, DEAE-Toyopearl 650M, and Toyopearl HW	ACG (Ligand free)/1WW7	1.90	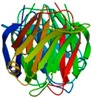 1WW7	Recombinant (rAAL-galectin)/2ZGL	[60,173,174]
		ACG Lac complex/1WW6	2.20			
		ACG 3'-sulfonyl Lac complex/1WW5	2.20			
		ACG α2,3-sialyllactose complex/1WW4	2.30			
		ACG mutant (N46A) blood type A antigen tetraose complex/3WG4	1.60			
		ACG blood type A antigen tetraose complex/3WG3	1.35			
*Coprinus cinereus* lectin (CGL2)	Lactosyl-sepharose AC	CGL2 (Ligand free)/1UL9	2.22	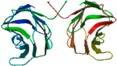 1UL9	CGL3 chitotetraose complex/2R0H	[82,175,176]
		CGL2 Lac complex/1ULC	2.60			
		CGL2 xeno linear trisaccharide complex/1ULE	2.15			
		CGL2 blood group A tetrasaccharide complex/1ULF	2.36			
		CGL2 TF antigen complex/1ULG	2.20			
		CGL2 blood H Type II complex/1ULD	2.20			
		CGL2 *C. elegans N*-glycan complex/2WKK	2.10			
*Coprinopsis cinerea* lectin (CGL3)	Lactosyl-sepharose AC	CGL3 (Ligand free)/2R0F	2.00	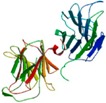 2R0F	CGL2 *C. elegans N*-glycan complex/2WKK	[82,84]
		CGL3 chitotetraose complex/2R0H	1.90			
**β-Trefoil fold**						
*Boletus edulis* lectin (BEL)	DEAE cellulose, Superdex G75, MonoQ, Lipidex 1000	BEL (Ligand free) form 1/4I4O	1.12	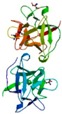 4I4P	LSL *N*-acetyllactoseamine complex/1W3F	[177]
		BEL (Ligand free) form 2/4I4P	1.28			
		BEL (Ligand free) form 3/4I4Q	1.51			
		BEL (Ligand free) form 4/4I4R	1.77			
		BEL lactose complex/4I4S	1.40			
		BEL galactose complex/4I4U	1.57			
		BEL *N*-acetylgalactosamine complex/4I4V	1.50			
		BEL T-Antigen disaccharide complex/4I4X	1.72			
		BEL T-Antigen complex/4I4Y	1.90			
*Clitocybe nebularis* lectin (CNL)	Lactosyl and glucosyl-sepharose AC, Chromsep HPLC	CNL Lac complex at pH 4.4/3NBC	1.01	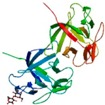 3NBC	Three Foil/3PG0	[79,81]
		CNL Lac complex at pH 7.1/3NBD	1.15			
		CNL *N*,*N*'-diacetyllactosediamine complex/3NBE	1.86			
*Coprinopsis cinerea* lectin (CCL2)	Horseradish Peroxidase AC	CCL2 (Ligand free)/2LIE	NA	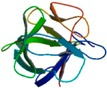 2LIE	Mosquitocidal toxin/2VSE	[83]
		CCL2 nematode glycan complex/2LIQ				
*Laetiporus sulphureus* lectin (LSL)	Lactose-sepharose AC	LSL (Ligand free)/1W3A	2.65	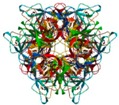 1W3A	*Boletus edulis* lectin (BEL)/4I4O	[76,111,112]
		LSL *N*-acetyllactoseamine complex/1W3G	2.68			
		LSL *N*-acetyllactoseamine complex in the Gamma motif/1W3F	2.58			
		LSL (recombinant)/2Y9F	1.47			
		LSL (recombinant) Lac complex/2Y9G	1.67			
*Marasmius oreades* lectin (MOA)	(NH_4_)_2_SO_4_ precipitation, melibiose-sepharose, Synsorb-type B trisaccharide, and Synsorb-type A trisaccharide AC	MOA Galβ1,3Galβ1,4-GlcNAc complex/2IHO	2.41	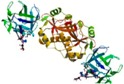 2IHO	Three Foil/3PG0	[122,178,179]
		MOA Galα1,3(Fucα1,2)Gal and calcium complex/3EF2	1.80			
*Macrolepiota procera* (MPL)	Lactosyl-Sepharose AC	MPL (ligand free)/4ION	1.60	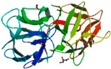 4ION	*Rhizoctonia solani* agglutinin/4G9M	[120]
		MPL Gal complex/4IYB	1.59			
		MPL Lac complex/4IZX	1.1			
		MPL *N*-acetyllactoseamine complex/4J2S	1.4			
*Polyporus squamosus* lectin (PSL)	(NH_4_)_2_SO_4_ precipitation, β-d-galactosyl-Synsorb AC, DEAE-Sephacel	PSL bound to human-type influenza-binding epitope Neu5Ac α2-6Galβ1-4GlcNAc/3PHZ	1.70	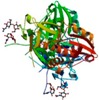 3PHZ	ThreeFoil/3PG0	[145,180]
**Actinoporin-like fold**						
*Agaricus bisporus* lectin (ABL)	Human erythrocytic stroma polyacrylamide gel AC, preparative isoelectric focusing to separate the 5 ABL isoforms	ABL (Ligand free)/1Y2T	1.50	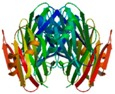 1Y2T	*Sclerotium rolfsii* lectin (SRL)/2OFC	[51]
		ABL Lacto-*N*-biose complex/1Y2U	1.85			
		ABL T-antigen complex/1Y2V	1.90			
		Orthorhombic form of ABL T-antigen and GlcNAc complex/1Y2W	1.74			
		Tetragonal form of ABL T-antigen and GlcNAc complex/1Y2X	2.36			
*Boletus edulis* lectin (BEL)	Chitin-sepharose AC, Superdex G-200 HR, Lipidex 1000; Human erythrocytic stroma polyacryl-amide gel AC, Superdex G-200 HR, Lipidex 1000 resin, MiniQ PE	BEL (Ligand free)/3QDS	1.15	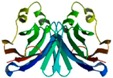 3QDS	*Sclerotium rolfsii* lectin (SRL)/2OFC	[177]
		BEL T-antigen complex/3QDT	1.30			
		BEL *N*,*N*-diacetyl chitobiose complex/3QDU	2.00			
		Orthorhombic form of BEL GlcNAc and GalNAc complex/3QDV	1.30			
		Hexagonal form of BEL GlcNAc and GalNAc complex/3QDW	1.90			
		Orthorhombic form of BEL T-antigen disaccharide and *N*,*N*-diacetyl chitobiose complex/3QDX	1.70			
		Hexagonal form of BEL T-antigen disaccharide and *N*,*N*-diacetyl chitobiose complex/3QDY	2.00			
*Xerocomus chrysenteron* lectin (XCL)	Fetuin-sepharose	Wild-type XCL (ligand free)/1XI0	2.00	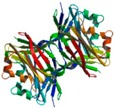 1XI0	*Sclerotium rolfsii* lectin (SRL)/2OFC	[107,181]
		XCL mutated at Q46M, V54M, L58M/1X99	1.40			

* For further lectin-related tools and databases please see the Lectin 3D structure database (http://glyco3d.cermav.cnrs.fr) or the CAZy database (http://cazy.org); ^ AC, Affinity Chromatography; ^#^ Only symmetrical, ligand free structures are shown; NA, Not Available.

**Table 3:** Antiproliferative/antitumor and mitogenic activity of mushroom lectins.

Antiproliferative/Antitumor Activity			
Source of Lectin	IC_50_	Cell Type/Target	Ref.
*Agaricus bisporus* (ABL)	50 µg/mL *	HT-29	[50]
*Agrocybe aegerita* (AAL galectin)	NA	S-180, Hela, SW480,SGC 7901, MGC80-3, BGC-823, HL-60	[56]
*Amanita phalloides* (*A. phalloides* lectin)	1.7 µg/mL *	L1210	[66]
*Armillaria luteovirens* (ALL)	2.5 μM	MBL2	[69]
	5 μM	HeLa	
	10 μM	L1210	
*Boletopsis leucomelaena* (BLL)	15 µg/mL	U937	[182]
*Clitocybe nebularis* (CNL)	NA	Mo-T, Jurkat	[79]
*Cordyceps militaris* (CML)	0.5–0.6 mg/mL *	HepG2	[183]
*Flammulina velutipes* (*F. velutipes* lectin)	13 μM	L1210	[86]
*Ganoderma capense* (*G. capense* lectin)	8 μM	L1210	[88]
	16.5 μM	HepG2	
	12.5 μM	M1	
*Grifola frondosa* (GFL)	25 μg/mL *	HeLa	[184]
*Hericium erinaceus* (HEA)	56.1 μM	HepG2	[94]
	76.5 μM	MCF7	
*Inocybe umbrinella* (*I. umbrinella* lectin)	3.5 μM	HepG2	[99]
	7.4 μM	MCF7	
*Lactarius flavidulus* (LFL)	8.90 μM	HepG2	[106]
	6.81 μM	L1210	
	7.4 μM	MCF7	
*Paecilomyces japonica* (PJA)	NA	SNU-1, AsPc-1, MDAMB-231	[130]
*Paxillus involutus* (*P. involutus* lectin)	NA	A-549, HCT-8	[185]
*Pholiota adiposa* (PAL)	2.1 μM	HepG2	[133]
	3.2 μM	MCF7	
*Pleurotus citrinopileatus* (*P. citrinopileatus* lectin)	5 mg/kg of body weight/day ^¥^	S-180 in ICR mice	[137]
*Pleurotus eous* (PEL)	2 μg/mL	MCF-7, K562, HEP-2	[139]
	50 μg/mL	SK-N-MC	
*Pleurotus ostreatus* (POL)	1.5 mg/kg bodyweight/day ^¥^	S-180, H-22	[32]
*Polyporus adustus* (*P. adustus* lectin)	NA	M1, Herto, S180	[144]
*Psathyrella asperospora* (PAL)	0.48 μM	HT29	[186]
*Russula delica*	0.88 μM	HepG2	[154]
	0.52 μM	MCF 7	
*Schizophyllum commune* (SCL)	30 μg/mL	KB	[156]
*Tricholoma mongolicum* (TML1 & TML2)	NA	P815, PU5-1.8	[187]
*Volvariella volvacea* (VVL)	17.5 mg/kg body weight ^¥^	S-180	[188]
*Xerocomus chrysenteron* (XCL)	NA	Hela, NIH-3T3	[189]
*Xylaria hypoxylon* (*X. hypoxylon* lectin)	1.24 μM	M1	[162]
	NA	HepG2	
**Mitogenic Activity**			
**Source of lectin**	**[Lectin] ^£^**	**Cell type/Target**	**Ref.**
*Agrocybe cylindracea* (ACG)	2 µM	Mouse splenocytes	[61]
*Armillaria luteovirens* (ALL)	1 µM	Mouse splenocytes	[69]
*Boletus edulis* (BEL)	1 µM	Mouse splenocytes	[72]
*Cordyceps militaris* (CML)	26 µM	Mouse splenocytes	[85]
*Flammulina velutipes* (*F. velutipes* lectin)	100 µM	Mouse spleen lymphocytes	[86,190]
*Hericium erinaceus* (HEA)	20 µM	Mouse splenocytes	[94]
*Ganoderma capense* (*G. capense* lectin)	1.5 µM	Mouse splenocytes	[88]
*Hygrophorus russula* (HRL)	0.15 µM	Mouse splenocytes	[97]
*Peziza silvestris* (*P. silvestris* lectin)	8 µM	Mouse splenocytes	[131]
*Pleurotus citrinopileatus* (*P. citrinopileatus* lectin)	2 µM	Mouse splenocytes	[137]
*Polyporus adustus* (*P. adustus* lectin)	62.5 µM	Mouse splenocytes	[144]
*Schizophyllum commune* (SCL)	4 µM	Mouse splenocytes	[157]
*Xerocomus spadiceus* (*X*. *spadiceus* lectin)	31.25 µM	Mouse splenocytes	[161]

NA: Not Available; * The concentration or range of titer found effective for antiproliferative/antitumor activities; ^¥^ The dosage found effective for *in vivo* antiproliferative/antitumor activities; ^£^ This is the minimum lectin concentration for mitogenic activity.

**Table 4:** Anti-HIV-1 reverse transcriptase activity of mushroom lectins.

Source of Lectin	IC_50_	Ref.
*Agaricus bisporus* (ABL)	8.0 µM	[191]
*Boletus edulis* (BEL)	14.3 µM	[72]
*Cordyceps militaris* (CML)	10.0 µM	[183]
*Hericium erinaceus* (HEA)	31.7 µM	[94]
*Inocybe umbrinella* (*I. umbrinella* lectin)	4.7 µM	[99]
*Pleurotus citrinopileatus* (*P. citrinopileatus* lectin)	0.93 µM	[137]
*Schizophyllum commune* (SCL)	1.2 µM	[157]

## 3. Mushroom Lectin Structures

Mushroom lectins usually comprise two to four identical or non-identical subunits held together by non-covalent interactions. However, at least two examples exist of mushroom lectin subunits being linked by disulphide bridges (*Lactarius lignyotus* [107] and *Phallus impudicus* [192]). The molecular mass and oligomeric state of isolated mushroom lectins vary greatly, ranging from 10 to 190 kDa [19]. Some notable examples include the tetrameric lectin with identical 16 kDa subunits from *Agaricus blazei* [53], the 12.4 and 18 kDa lectins isolated from the *Ganoderma lucidum* mycelia and fruiting bodies respectively [88], the 23 kDa monomeric lectin from *Auricularia polytricha* [70], and the 114 kDa hexameric lectin form *Ganoderma capense* [89,90]. The most commonly used methods for the purification of mushroom lectins are ion-exchange chromatography, affinity chromatography based on the sugar specificity of the lectin of interest, and size-exclusion chromatography, with the order and number of purification steps varying from lectin and lectin. Table 2 summarises the common strategies used to purify mushroom lectins for which crystal structure data exists.

In order to better understand the atomic structure and molecular mechanisms underlying lectin-sugar interactions, it is necessary to study their crystallographic structure. The crystal structures of only 17 mushroom lectins have been determined (Table 2). A number of different structural families of mushroom lectins have been identified, including the ricin-like β-trefoil fold, galectin-like fold and actinoporin-like fold. The integrin-like seven-blade β-propeller and six-blade β-propeller as well as the actinoporin-like fold are distinctive for lectins from fungi [44,193]. Table 2 summarises the common structural characteristics mushroom lectins for which crystal structure data exists.

The crystal structures of two mushroom lectins have been reported to possess β-propeller-folds, the *Aleuria aurantia* lectin (AAL) and the *Psathyrella velutina* lectin (PVL). Among all mushroom lectins, PVL is the best studied. PVL is a multivalent and multi-substrate specific lectin that adopts a regular seven-bladed β-propeller-fold. The structure of PVL complexed with GlcNAc revealed six residues bound in pockets located between two consecutive propeller blades. A complex of PVL with Neu5Ac showed that the same hydrogen bond network as seen for GlcNAc are present, but the carbohydrate ring in the binding site is oriented differently [169]. The crystal structure of AAL complexed with Fuc revealed that each of the two monomers that make up AAL consist of a six-bladed β-propeller fold and a small antiparallel two-stranded β-sheet that is involved in dimerization. Interestingly, AAL was found to possess a multivalent carbohydrate recognition fold [166], similar to that seen in the Fuc binding lectin from *Aspergillus fumigatus* (AFL1), which has been proposed to be involved in the host pathogen interaction [194]. Structural PVL and AAL resemble α-integrins, and are very similar to the bacterial lectin RSL from *Ralstonia solanacearum* that also adopts a β-propeller fold [195].

As previously mentioned a number of mushroom lectins have been found to adopt a galectin, or galectin-like fold. Galectins have a conserved carbohydrate recognition domain (CRD) that shares a β-Gal recognition pattern with highly conserved side chains. The galectins reported for *Agrocybe aegerita* (designated here AAL-galectin to distinguish it from the *Aleuria aurantia* lectin which is commonly referred to in the literature as AAL), *Agrocybe cylindracea* (ACG) and *Coprinus cinereus* (CGL2 and CGL3) belong to the proto-type galectin family. Based on sequence and structural comparison AAL-galectin and ACG are identical. The crystal structure of ACG complexed with Lac, 3'-sulfonyl Lac, and α2,3-sialyllactose revealed a β-sandwich structure of two antiparallel sheets, each with six strands, in contrast to the five and six strands in animal galectins [173]. Except for the substitution of one residue the CRD is the same as that seen in animal galectins. Interestingly, the presence of a 5-residue insertion in ACG alters the carbohydrate-binding site such that it is able to bind Neu5Ac [173]. The structure of CGL2 [175] and CGL3 [84] have also been studied complexed with a number of ligands including Lac, linear B2 trisaccharide, blood group A tetrasaccharide, and blood H Type II for CGL2, and chitotetraose (GlcNAcβ1,4GlcNAcβ1,4GlcNAcβ1,4GlcNAc) for CGL3. CGL3 conserves all but one residue (Arg replaces Trp) known to be involved in Gal binding in galectins, resulting in CGL3 being unable to bind Lac. Instead CGL3 specifically and preferentially binds oligomers of GlcNAc. Interestingly, the mutation of Arg to Trp resulted in the lectin being unable to bind oligomers of GlcNAc and instead was able to bind Lac [84]. As will be discussed later in this review, a number of mushroom lectins have been found to have antitumour activity. One such example is AAL-galectin, which suppresses tumours through apoptosis-inducing activity in cancer cells [56]. The Thomsen-Friedenreich antigen (TF antigen; Galβ1-3GalNAc-*O*-Ser/Thr) is believed to be the ligand for AAL-galectin. The crystal structure of AAL-galectin complexed with the TF antigen revealed a unique recognition mode consisting of a hydrogen bond network formed by a conserved structural motif (Glu66-water-Arg85-water) that provides new targets and opportunities for anticancer drug discovery [171].

Ricin B-like (β-trefoil) lectins are carbohydrate-binding proteins similar to the B chain domains of ricin, a toxin from the castor bean (*Ricinus communis*) [196]. The main characteristic of these lectin domains is that they consist of three repeated subdomains, referred to as α-, β- and γ-repeats, each containing a well-conserved QXW motif [197]. Several ricin B-like lectins have been identified in mushrooms and crystal structures have been solved for *Clitocybe nebularis* lectin (CNL) [81], *Laetiporus sulphureus* lectin (LSL) [112], *Marasmius oreades* lectin (MOA) [178], *Polyporus squamosus* lectin (PSL) [180], *Coprinopsis cinerea* lectin (CCL2) [83], *Macrolepiota procera* (MPL) [120] and *Boletus edulis* lectin (BEL β-trefoil) [177]. LSL contains a pore-forming module [112], whereas a cysteine protease domain that also serves as a dimerization interface is present in MOA and PSL [178,180,198]. The toxic activities of these modular proteins have been attributed to their catalytic domains and the intracellular transport of the protein through binding to glycolipids or glycoproteins (Refer to Table 1 and Table 2 for exact sugar specificity) that are facilitated by lectin domains [198,199].

Another unique fold among the mushroom lectin is the actinoporin-like fold. The actinoporin-like fold consists of a β-sandwich made by two β-sheets composed of six and four β-strands respectively and connected by a helix-loop-helix motif. This fold has been found in *Agaricus bisporus* lectin (ABL) [51], *Boletus edulis* lectin (BEL) [177] and *Xerocomus chrysenteron* lectin (XCL) [181]. These lectins form dimers or tetramers that have two distinct binding sites per monomer that recognize the different configurations of a single epimeric hydroxyl.

*Lyophyllum decastes* lectin (LDL) is an interesting new addition to the known mushroom lectins. The recently resolved structure of LDL shows that this novel lectin adopts a unique lectin fold, where a core of two antiparallel β-sheets at the heart of a homodimer is connected to the periphery of the structure by intramolecular disulfide bridges [165]. Furthermore, the structure and LDL’s fold suggests that it is an extracellular protein unlike most known mushroom lectins.

## 4. Biological Activity of Mushroom Lectins

### 4.1. Antiproliferative/Antitumor Activity

Tumour cell surfaces vary in composition of glycoconjugates in comparison to normal cells [200]. Lectins display antiproliferative potential by cross-linking these cell surface glycoconjugates or through immunomodulatory effects. The Galβ1,3GalNAc-bindinglectin from the edible mushroom *Agaricus bisporus* inhibits growth of colon cancer cells and breast cancer [50]. Similarly, the *Volvariella volvacea* lectin that has antiproliferative activity against Sarcomo S-180 cells [19], has also been shown to retard the growth of tumour cells in a mouse model, prolonging the life span of mice by 63% to 100%. The lectin from *Grifola frondosa* is reported to be cytotoxic against HeLa cells at the lectin concentration of 25 μg/mL [184]. The antiproliferative activity of lectins has also been demonstrated in other mushroom species including *Paxillus involutus* [185], *Lactarius flavidulus* [106], *Hericium erinaceus* [94], *Russula delica* [154], *Pholiota adiposa* [133], and *Clitocybe nebularis* [79], and we have recently shown that the GlcNAc-specific lectin from *Psathyrella asperospora* (PAL) possess a potent antiproliferative activity (IC_50_: 0.48 μM) [186]. Further characterization of PAL’s anti-proliferative activity showed that HT29 cells are arrested at G2/M phase of the cell cycle, and that this effect can be halted through the addition of free GlcNAc. Table 3 summarises the antiproliferative and antitumor activity of mushroom lectins along with their potent activity toward cancer cell lines.

### 4.2. Mitogenic/Antimitogenic Activity

Some lectins possess the remarkable property of stimulating the transformation of lymphocytes from small resting cells to large blast-like cells that may undergo mitosis [201]. Furthermore, the mechanism of this mitogenic activity, which involves activation and proliferation of lymphocytes, usually commences by binding of ligands to T-cell receptors, which triggers the signaling cascade, IL-2 gene expression and subsequent proliferation [202]. Lectins from *Flammulina velutipes* [190], *Armillaria luteovirens* [69], *Ganoderma capense* [88], *Agrocybe cylindracea* [61], *Xerocomus spadiceus* [161], *Boletus edulis* [72], *Cordyceps militaris* [85], *Pleurotus citrinopileatus* [137] and *Hygrophorus russula* [97] are known to be mitogenic with respect to murine splenocytes. In addition, the *Volvariella volvacea* lectin possesses mitogenic activity towards T lymphocytes through T-cell receptor ensuing calcium signaling pathways [203]. The mitogenic potential of lectins frommushrooms is summarized in Table 3. However, lectins do not always display mitogenic activity to lymphocytes. Certain lectins have demonstrated antimitogenic activity. The lectin from *Agaricus bisporus* suppresses the activation of T and B lymphocytes [204]. Likewise, the lectins from *Pleurotus flabellatus* [203], *Hericium erinaceus* [203], *Tricholoma mongolicum* [45], *Laetiporus sulphureus* [113], *Lactarius deliciosus* [104], and *Xylaria hypoxylon* [162] are non-mitogenic.

### 4.3. Immunomodulatory Activity

There are only a few mushroom lectins that have been reported to regulate the components of the immune system. The lectins from *Tricholoma mongolicum* (TML-1 and TML-2) have been shown to activate macrophages through the generation of macrophage activating factor and tumor necrosis factor (TNF) in mice by stimulating the production of NO_2_ ions [187]. In a similar manner, ABL and ACG are also able to stimulate macrophage through the production of TNF-α and NO_2_ [205,206]. Interestingly, ABL’s immunomodulating activity was found to be thermal/freezing-resistant, acid/alkali tolerant and stable to dehydration making it a potential candidate as a stable immune stimulant in health foods and pharmaceuticals [205]. The lectin from *Volvariella volvacea* (VVL) also exerts a potent immunomodulatory effect in mice by inducing the gene expression of IL-2 and IFN-γ, thereby upregulating the Th-1 cell population [31]. In fact, VVL was nine-fold more potent than other non-lectin mushroom immunomodulating proteins in activating lymphocytes [207].

### 4.4. Antiviral Activity

The *Paxillus involutus* lectin possesses antiphytovirus activity towards tobacco mosaic virus with 70.6% inhibition at a concentration of 200 μg/mL, however does nothave any inhibitory activity towards HIV-1 reverse transcriptase [185]. *Hericium erinaceus* agglutinin (HEA) on the other hand is a demonstrated inhibitor of HIV-1 reverse transcriptase activity with an IC_50_ of 31.7 µM [94]. In fact a number of mushroom lectins havebeen found to havepotent anti-HIV-1 reverse transcriptase activity. For example, ABL, *Schizophyllum commune* lectin, BEL, *Pleurotus citrinopileatus* lectin, *Cordyceps militaris* lectin, and *Inocybe umbrinella* lectin, are all the inhibitors of HIV-1 reverse transcriptase [72,99,137,157,183,191]. The most potent anti-HIV-1 reverse transcriptase mushroom lectin yet identified comes from *Pleurotus citrinopileatus* (PCL) with an IC_50_ of 0.93 µM [137].

The exact mechanism by which lectins in general exert their anti-HIV-1 reverse transcriptase activity is yet to be fully resolved but probably involves protein–protein interaction as demonstrated for the HIV-1 protease that also inhibits HIV-1 reverse transcriptase activity [208]. Besides mushroom lectins, antifungal proteins [209], ribosome inactivating proteins [210], and plant lectins [211] have also been shown to inhibit HIV-1 reverse transcriptase. Interestingly, the well characterized lectins Concanavalin A and ricin, and the red kidney bean agglutinin are potent inhibitors of DNA polymerase alpha activity, and DNA polymerase beta activity respectively [212]. The significance of this activity in relation to reverse transcriptase inhibition by other lectins awaits elucidation.

In addition to mushroom lectins, other fungal lectins also possess anti-HIV activity such as the cyanovirin-*N* homologs (CVNHs) from *Ceratopteris richardii* and *Neurospora crassa* [213]*.* However these lectins were found not to be as effective as the native bacterial cyanovirin-*N* and other known anti-viral lectins including griffithsin, scytovirin and microcystis virdis lectin [214]. The activity of cyanovirin-*N* primarily arises from its association with the viral envelope glycoprotein 120 and to other cell surface receptors [215,216]. Again the significance and relevance of this mechanism in relation to anti-HIV activity shown by other lectins awaits elucidation.

## 5. Conclusions and Future Perspectives

The ability of lectins to bind specifically to glycoconjugates present on the cell surface has made them essential tools in diverse applications. For example, taxonomic study of protozoan parasites has been performed using the agglutinating extracts from several macrofungi [217,218]. In biomedical research, purified lectins are used to determine blood type due to the specificity of carbohydrate structures present on the cell surface of erythrocytes. Among the mushroom lectins, MOA has been reported to be specific for blood group B, while CGL2 is specific for blood group A tetrasaccharide [173,179]. Interestingly, the use of the *Pleurotus ostreatus* lectin (POL) as an adjuvant in hepatitis B virus (HBV) DNA vaccination has been reported to stimulate the immune response in transgenic mice. It has also been demonstrated that low dose of POL (1 µg/mouse) in conjunction with HBV DNA vaccine has given stronger HBV-specific delayed-type hypersensitivity responses and higher HBV-specific IgG levels in that particular transgenic mice [219].

Another practical use of lectins is based on their immobilization to an inert chromatography support, which has allowed the isolation of particular membrane and serum glycoconjugates [40]. For example, AAL has been used to isolate different glycoconjugates, such as fucosylated glycoproteins from human erythrocyte membrane [220], brain glycoproteins [221], Bence-Jones proteins [222], tumour antigens [223], and human immunoglobulin G [224]. Furthermore, the specificity of lectins for sugars makes them valuable tools in glycobiology. For example, lectin arrays (immobilised lectins with known sugar binding specificity covalently immobilised onto glass microarray slides) are a relatively new tool in glycobiology that can be used to analyse the glycosylation pattern on the surface of cells, and, thus, determine its relevance in various cell processes including cell development and differentiation, cell-cell communication and pathogen-host recognition [225].

Despite the diversity of bioactivity attributed to lectins, there is only limited information available regarding the relationship between structure, function and biological activity of mushroom lectins. Recently, an attempt was made to structurally determine the correlation between tumour cell apoptosis-induction and *Agrocybe aegerita* lectin activity. It was reported that the prerequisite for tumour cell apoptosis inducedby *Agrocybe aegerita* lectin was the formation of dimers, and that binding of both Gal and Glc are required for lectin bioactivity [172]. Similarly, Pohleven *et al.* demonstrated that the carbohydrate binding and homodimerization properties of CNL, a ricin B-like lectin from *Clitocybe nebularis*, was essential for bioactivity, with non sugar-binding and nondimerizing monovalent CNL mutants being inactive [81]. Further studies are required to determine the mechanism of action that gives mushroom lectins, and lectins from other sources, their diverse and potent bioactivity.

The potential use of mushroom lectins in therapy will also require the large-scale production of pure, fully functioning protein. Currently the majority of potentially medical useful mushroom lectins are purified from fruit-bodies collected in nature. This not only gives low yields, but is also time-consuming and expensive, and can also lead to batch variation. Moreover, the native isolation of lectins from mushrooms can lead to batch variation, due to environmental influences such as season, location and year of harvest, as well as differences in mushroom maturity and mycelia growth [7,40]. Therefore there is a need to advance efforts to clone and recombinantly express functional lectins. Over the past decade, only a small number of mushroom lectins have been expressed in *Escherichia coli*. For example, AAL and AAL2 lectin from the edible mushroom *Agrocybe aegerita* have been cloned and functionally expressed in *E. coli* [59,172]. In addition, CNL [81], MOA [178], XCL [181], and PSL [180] have also all been expressed in *E. coli*, and CGL2 has been expressed in the yeast *Saccharomyces cerevisiae* [175]. However, the majority of these lectins have been recombinantly expressed to obtain lectin in high enough yields for crystal structure determination. Recombinant lectin production for potential therapeutic use is still in its infancy, and will require further research and development particularly with regard to the importance and requirements for post-translational modifications of lectins for therapeutic use in humans.
